# Excellent Response to Atezolizumab After Clinically Defined Hyperprogression Upon Previous Treatment With Pembrolizumab in Metastatic Triple-Negative Breast Cancer: A Case Report and Review of the Literature

**DOI:** 10.3389/fimmu.2021.608292

**Published:** 2021-05-31

**Authors:** Dongfeng Feng, Yaping Guan, Mingguo Liu, Shuqian He, Weipeng Zhao, Beibei Yin, Jing Liang, Yan Li, Jun Wang

**Affiliations:** ^1^ Department of Oncology, The First Affiliated Hospital of Shandong First Medical University & Shandong Provincial Qianfoshan Hospital, Jinan, China; ^2^ Shandong Lung Cancer Institute, Jinan, China; ^3^ Shandong Key Laboratory of Rheumatic Disease and Translational Medicine, Jinan, China; ^4^ Department of Oncology, Yuncheng Honesty Hospital, Heze, China; ^5^ Department of Pathology, The First Affiliated Hospital of Shandong First Medical University, Jinan, China; ^6^ Department of Breast Cancer, Tianjin Medical University Cancer Institute and Hospital, National Clinical Research Center for Cancer, Key Laboratory of Cancer Prevention and Therapy, Tianjin, China

**Keywords:** hyperprogressive disease, immune checkpoint inhibitor, pembrolizumab, atezolizumab, triple-negative breast cancer

## Abstract

Immunotherapy with immune checkpoint inhibitors (ICIs), including programmed cell death protein-1 (PD-1) and programmed cell death ligand-1 (PD-L1) inhibitors, has revolutionized the systematic treatment of advanced and metastatic solid tumors. However, the response rate to ICIs is unsatisfactory, and unexpected hyperprogressive disease (HPD) is even observed in a small subgroup of patients. Patients with HPD usually have worsening clinical symptoms and poorer survival, and therapeutic strategies are extremely limited. Here, we presented a patient with HPD who had used a PD-L1 inhibitor and was highly responsive to the sequential use of a PD-1 inhibitor. A 67-year-old woman with metastatic triple-negative breast cancer was treated with pembrolizumab plus chemotherapy after progression on previous multiple-line chemotherapy treatments. After 2 cycles of treatments, she rapidly developed HPD, as confirmed by radiological evaluation and worsening symptoms. At that time, pembrolizumab was discontinued, and she switched to the PD-L1 inhibitor atezolizumab plus chemotherapy. This patient partially responded to atezolizumab plus chemotherapy without experiencing severe drug-related adverse effects. This is the first reported case of metastatic breast cancer in a patient with radiologically confirmed HPD after pembrolizumab therapy in which successful rechallenge with atezolizumab relieved clinical symptoms. Further studies with larger sample sizes involving a deeper translational investigation of HPD are needed to confirm the efficacy and mechanism of sequential application of different ICIs for the clinical management of HPD.

## Introduction

Immunotherapy with immune checkpoint inhibitors (ICIs), including programmed cell death protein-1 (PD-1) and programmed cell death ligand-1 (PD-L1) inhibitors, has revolutionized the systematic treatment of advanced and metastatic human solid tumors (1). ICIs selectively restore and normalize the body’s immune responses by disrupting the immunoinhibitory signals mediated by PD-1, PD-L1 and cytotoxic T-lymphocyte-associated protein 4 (CTLA-4) in the tumor microenvironment (2). Considering that they can achieve durable tumor remission, are well tolerated and have manageable toxicity in terms of immune-related adverse events (irAEs), ICIs have been approved for the treatment of advanced non-small cell lung cancer (NSCLC), melanoma, and various other human cancers.

The response to ICI-based monotherapy is limited, with an objective response rate of 20-30% (3). Furthermore, unexpected response patterns usually occur in a subgroup of patients at a low frequency following immunotherapy, including pseudoprogression, hyperprogressive disease (HPD), and mixed response (4). In particular, HPD has recently been reported in patients with various types of tumors, including NSCLC (5), head and neck squamous carcinoma (6), gastric cancer (7), melanoma (8), and hepatocellular carcinoma (9), which may be attributable to the increased application of ICIs and improved recognition of different tumor progression patterns. Generally, HPD is characterized by aggressive progression at an accelerated and unexpected rate and increased tumor burden within a short-term period. Patients with HPD usually experience worsening symptoms, poor performance status and poor survival, and therapeutic strategies are extremely limited (10). Elucidation of the mechanisms associated with HPD and the development of medicines that can treat patients with HPD are urgently needed. Here, we described one case of metastatic breast cancer in a patient with HPD initially induced by the PD-1 inhibitor pembrolizumab. The patient subsequently switched to the PD-L1 inhibitor atezolizumab plus chemotherapy. The patient exhibited a partial response to atezolizumab plus chemotherapy without experiencing severe drug-related adverse effects.

## Case Presentation

**Figure 1:**
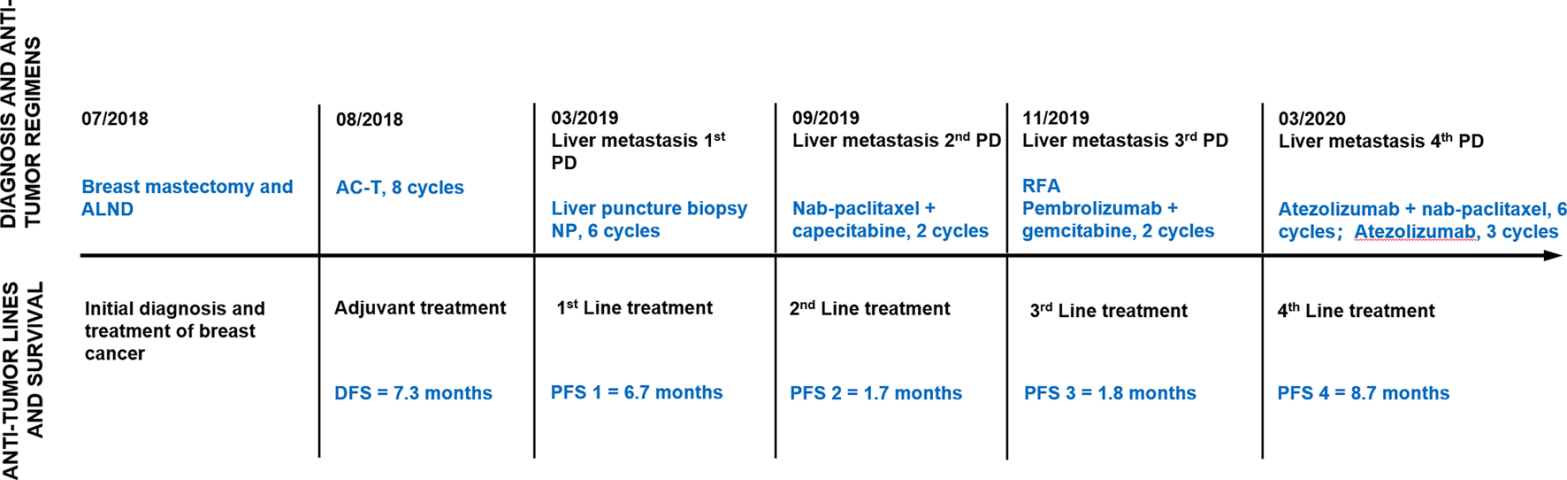
Timeline of antitumor regimens and outcomes. Line graph illustrating disease progression, antitumor lines, regimens, and outcomes from July 2018 to November 2020. ALND, axillary lymph node dissection; AC-T, doxorubicin, cyclophosphamide followed by paclitaxel; PD, progressive disease; NP, vinorelbine plus carboplatin; RFA, radiofrequency ablation; DFS, disease-free survival; PFS, progression-free survival.

In July 2018, a 67-year-old Chinese woman was referred to the First Affiliated Hospital of Shandong First Medical University because of a 2-month history of a single palpable mass in her left breast. Her medical history was not remarkable. She was initially diagnosed with ductal carcinoma of the left breast through fine-needle biopsy for this mass. She underwent left mastectomy and axillary node dissection, and a pathological report revealed a diagnosis of infiltrating ductal carcinoma, estrogen receptor (ER)-negative, progesterone receptor (PR)-negative, and HER2-negative (2+ by IHC and FISH negative) by immunohistochemistry (IHC). She was recognized as having stage III triple-negative breast cancer (TNBC) according to the American Joint Committee on Cancer Staging Manual, 7th edition. She received 8 cycles of adjuvant chemotherapy with doxorubicin and cyclophosphamide followed by paclitaxel (AC-T) with tolerable adverse effects but refused to receive radiotherapy (**Figure 1**).

**Figure 2:**
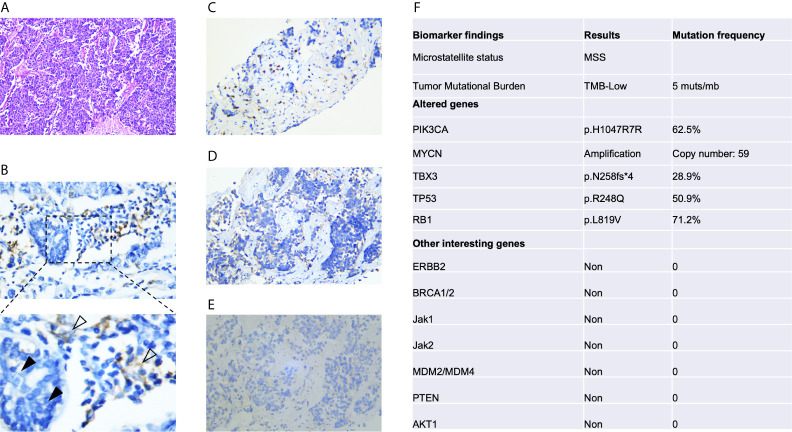
Immunohistochemical analysis of PD-L1 expression and next-generation sequencing of pretreatment tumors. **(A)** Hematoxylin-eosin staining. **(B)** Immunohistochemical analysis of PD-L1 expression was conducted with an anti-PD-L1 antibody (Dako PD-L1 22C3 PharmDx assay). PD-L1 was not expressed in tumor cells (black arrow), whereas positive staining for PD-L1 in peritumoral lymphocytes was observed (white arrow). Immunohistochemical detection of CD3+ **(C)**, CD4+ **(D)** and CD8+ cells **(E)**. **(F)** Next-generation sequencing results based on the FoundationOne CDx assay. The test analyzes 324 genes as well as genomic signatures, including microsatellite instability and tumor mutation burden.

In March 2019, this patient was admitted to Qilu Hospital, Cheeloo College of Medicine, Shandong University for regular follow-up. Computed tomography (CT) showed multiple metastatic lesions in the liver, with the largest lesion measuring 1.1 × 1.0 cm. There was no direct pain or other symptoms associated with these masses. She underwent liver fine-needle aspiration, and the pathology was confirmed as metastatic TNBC (**Figure 2A**). Subsequently, she was administered systematic multiple-line treatments, including vinorelbine plus carboplatin and nab-paclitaxel plus capecitabine. Unfortunately, her disease ultimately progressed while on these systematic therapies, although some lesions shrank within a short period of time.

In September 2019, she was again referred to our hospital with abdominal pain and severe fatigue. A magnetic resonance imaging (MRI) scan showed that her disease had further worsened, as extensive and large lesions were observed in the liver, but there was no tumor spread to the brain, lungs, and bone (**Figure 2A**). This patient had an Eastern Cooperative Oncology Group score of 1. Laboratory testing, including routine blood tests, biochemical tests, and urinalysis, revealed normal or negative results, but she had elevated alanine transaminase (74 U/L), aspartate transaminase (71 U/L), alkaline phosphatase (267 U/L), and creatinine (105 μmol/L) levels. A solid tumor gene profiling test by next-generation sequencing was conducted by the FoundationOne CDx assay, which showed that the tumor had a low tumor mutational burden (TMB) of 5 mutants per megabase (muts/mb), positive PI3KCA mutation (p. H1047R), and MYCN amplification. Her tumor did not harbor BRCA1/2 mutations, other homologous recombination deficiencies, or potential mutations associated with HPD (**Figure 2F**).

**Figure 3:**
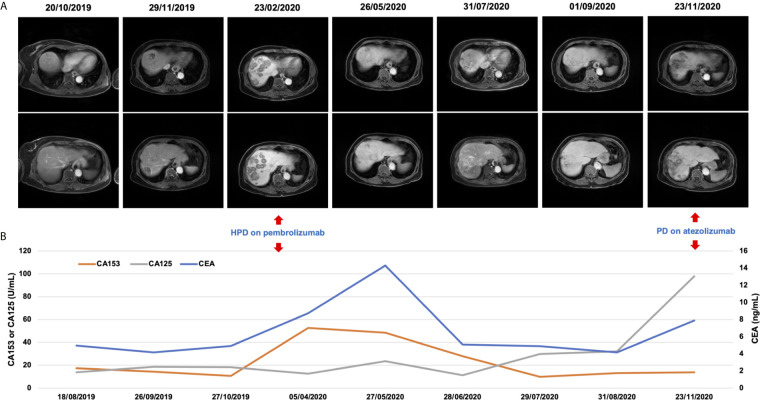
Radiological evaluation and altered levels of circulating tumor markers before and after HPD. **(A)** After receiving immunotherapy with pembrolizumab, this patient developed HPD. An MRI scan showing more extensive and large metastatic lesions in the liver, which responded well to rechallenge with atezolizumab in May 2020, and necrotic areas. **(B)** Dynamics of the levels of circulating tumor markers (CEA, CA125, and CA153) before and after HPD.

**Figure 4:**
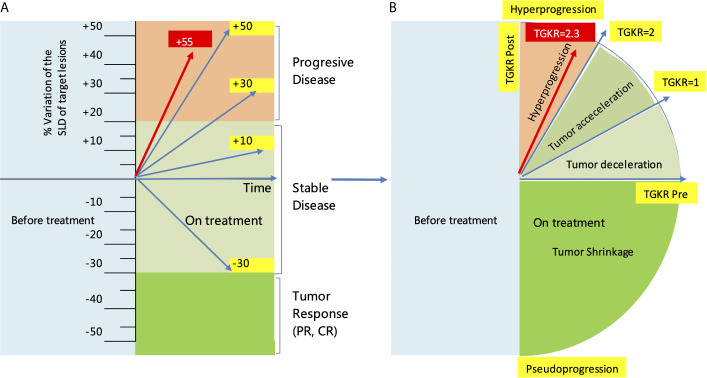
Evaluation of HPD by the RECIST1.1 criteria and tumor growth kinetics after starting immunotherapy with pembrolizumab. **(A)** Evaluation of HPD by measuring the variation in the SLD of target lesions according to RECIST1.1 criteria. **(B)** Evaluation of HPD by measuring the TGKR. SLD, sum of the longest diameters; TGKR, tumor growth kinetics ratio; HPD, hyperprogressive disease; RECIST, response evaluation criteria in solid tumors.

**Figure 5:**
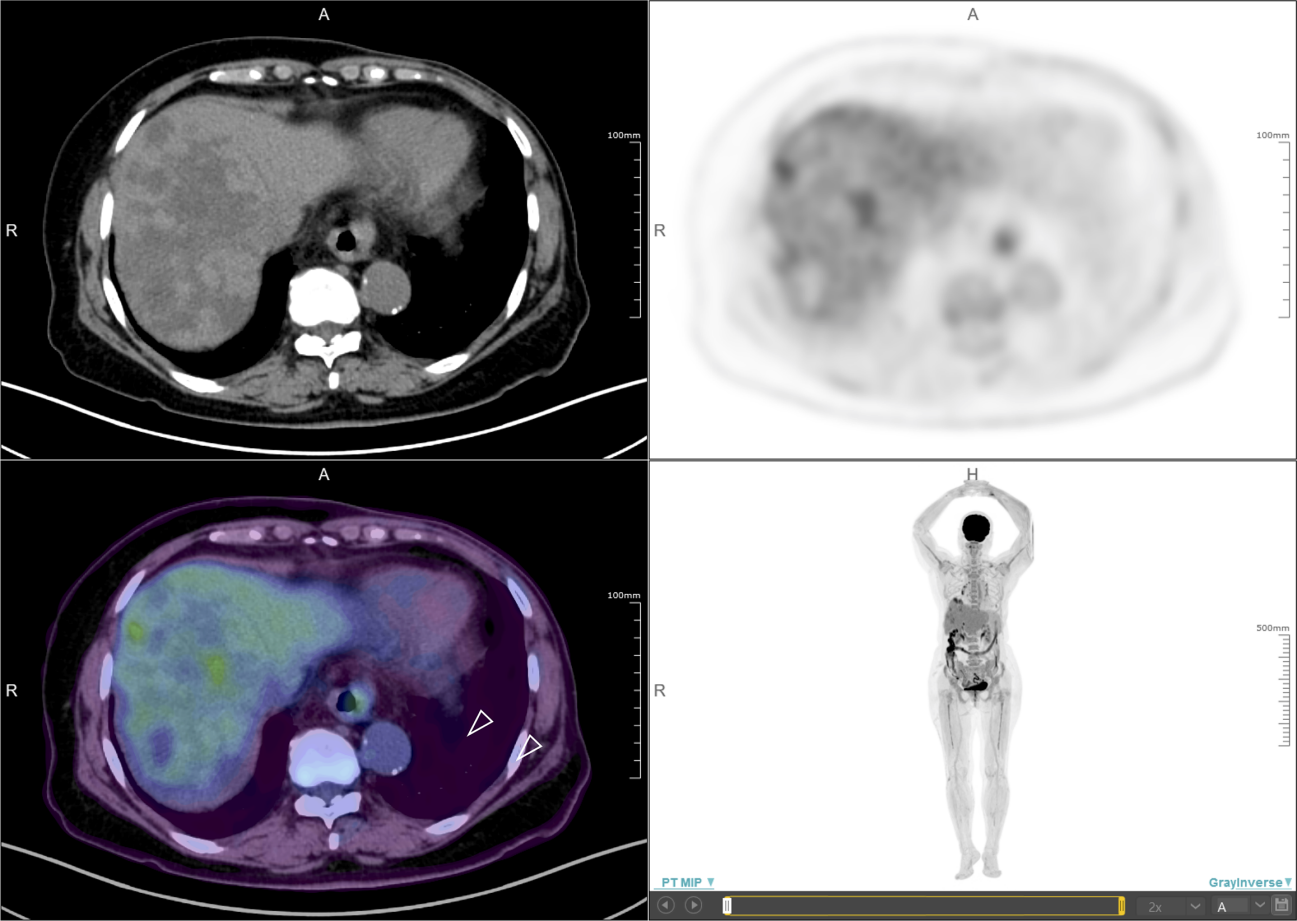
Positron emission tomography/computed tomography (PET/CT). PET/CT scan confirmed slightly high fluorodeoxyglucose (^18^F-FDG) uptake with an SUV_max_ of 5.9 for limited metastatic liver lesions (white arrow).

In November 2019, she received partial radiofrequency ablation treatment for her specific metastatic liver lesion. In January 2020, she received third-line treatment with gemcitabine plus the PD-1 inhibitor pembrolizumab (200 mg every 3 weeks). Following 2 cycles of treatments, the previous lesions in the liver rapidly progressed, new lesions occurred and the levels of CEA and CA153 were increased (**Figure 3B**). She also had some obvious clinical presentations, including moderate fatigue, loss of appetite, abdominal pain, and abdominal tenderness. No irAEs were recorded after immunotherapy. HPD was confirmed through MRI scanning according to a previously reported definition of HPD in terms of tumor burden (**Figure 4A**) and the tumor growth kinetics ratio (TGKR) (**Figure 4B**). Briefly, HPD is defined as a greater than 50% increase in tumor burden compared to previous imaging and a > 2-fold increase in the TGKR, which is calculated by comparing TGK to that on immunotherapy (11). At that time, IHC using the PD-L1 IHC 22C3 pharmDx assay showed a tumor cell PD-L1 expression percentage of 0% and immune cell PD-L1 expression of 5% (Dako, Inc.) (**Figure 2B**). CD3+ cells were visible, but CD4+ and CD8+ cells were absent (**Figures 2C–E**). Considering that the IMpassion130 trial revealed that atezolizumab plus nab-paclitaxel is superior to placebo plus nab-paclitaxel in unresectable locally advanced or metastatic TNBC (12) and that atezolizumab was available in China at that time, a fourth-line treatment with nab-paclitaxel plus the PD-L1 inhibitor atezolizumab (200 mg every 3 weeks) was started in March 2020. At the time of the first radiological tumor reassessment, the lesions in her liver had responded well to this regimen, with a significant objective partial response (**Figure 3A**). A positron emission tomography/computed tomography (PET/CT) scan confirmed slightly high fluorodeoxyglucose (^18^F-FDG) uptake with an SUV_max_ of 5.9 for limited metastatic liver lesions (**Figure 5**). Unexpectedly, the treatment reduced alanine transaminase, aspartate transaminase, alkaline phosphatase, creatinine, CEA, and CA125 levels to normal levels (**Figure 3B**). Except for grade 1 pruritus and grade 2 fatigue, she tolerated immunotherapy plus chemotherapy well. She continued to receive atezolizumab until November 2020, when the lesions in her liver progressed again (**Figure 3A**). She maintained a partial response to rechallenge with atezolizumab for more than 8 months.

## Discussion

### The Incidence and Definition of HPD

HPD is characterized as a new aggressive pattern of cancer progression and has previously been reported in patients receiving chemotherapy and targeted therapy. Nearly 8-29% of patients receiving PD-1/PD-L1 inhibitors develop HPD. Since 2016, HPD has been reported by multiple institutions in patients with melanoma (8), NSCLC (5), head and neck squamous carcinoma (6), hepatocellular carcinoma (9), and gastric cancer (7). Although there is no standard definition of HPD, HPD patients’ tumors progress at an accelerated and unexpected rate and increase in volume within a short period of time. HPD is most likely to occur in older patients (> 65 years old), patients with activating endothelial growth factor receptor (EGFR) mutations, patients with head and neck squamous carcinoma, patients with an elevated neutrophil-to-lymphocyte ratio, and patients with > 2 metastatic sites before PD-1/PD-L1 inhibitor therapy. A recently published meta-analysis showed that the occurrence of HPD was related to ECOG > 1, decreased serum levels of albumin, elevated serum levels of lactase dehydrogenase, and extensive liver metastases (13). However, positive PD-L1 expression status in these patients seems to be inversely correlated with HPD (OR = 0.60, *P* = 0.044) (14). The present patient showed clinically defined HPD following the application of pembrolizumab, with a time-to-treatment of less than 2 months, a 55% increase in tumor burden, and a 2.3-fold increase in the TGKR, which is completely in line with the clinical definition of HPD by Kato (11). Although pseudoprogression can sometimes be associated with radiological findings of accelerated tumor growth and is similar in appearance to HPD (15), pseudoprogression should not be considered when a patient’s symptoms continuously worsen. The patient also had some obvious positive symptoms and signs, including moderate fatigue, loss of appetite, abdominal pain, and abdominal tenderness. No irAEs were recorded after immunotherapy. This case report described clinically defined HPD for the first time in a patient with metastatic breast cancer treated with a PD-1 inhibitor. The initial multiple liver metastases in this 67-year-old patient could be a risk factor for HPD, or incomplete radiofrequency ablation could have promoted tumor progression and hindered the efficacy of anti-PD-1 therapy by inducing sustained local inflammation with predominant myeloid suppressor cells, which has been identified in clinical practice and mouse models (16).

### The Mechanism of HPD

Although the definitive cellular and molecular mechanism associated with HPD remains unclear, alterations in the levels of specific genes that lead to the activation of the corresponding signaling pathway of tumor growth may be attributable to HPD. MDM2/MDM4 amplification (11) and activation of AKT1 E17K have been found in HPD patients with immunotherapy (17). Furthermore, other gene alterations in posttreatment HPD tumors, including TSC2 and VHL mutations, are linked to the risk of HPD. Oncogenic pathways, including the IGF-1, ERK/MAPK, PI3K/AKT, and TGF-β pathways, are activated in HPD tumors after anti-PD-1 therapy (18). It is hypothesized that PD-L1 binding in itself might cause genetic alterations and subsequent increased tumor progression (19). In the present case, gene sequencing by the FoundationOne Dx assay indicated that pretreatment tumors did not harbor any previously reported mutations associated with HPD.

Immune cells inside the microenvironment could favor cancer cell invasion and accelerate progression. Clustered infiltrating epithelioid macrophages characterized by a CD163+CD33+PD-L1+ profile have been found in pretreatment tumors and contribute to HPD *via* the anti-PD-1 antibody-Fc/FcR interaction (20). Innate lymphoid cells 3 could be responsible for the occurrence of HPD because these cells can promote cancer development by secreting the cytokines IL-17, IL-22, and GM-CSF in an antigen-independent manner (21). Recently, posttreatment intratumoral proliferating Tregs (22), baseline and posttreatment highly differentiated human cells defined by a CD28-CD27-CD4+ phenotype (23), and TIGIT+ T cells among PD-1+CD8+ T cells (24) were shown to be activated in HPD tumors. Furthermore, incomplete radiofrequency ablation could promote tumor progression by inducing sustained local inflammation with predominant myeloid suppressor cells and the accumulation of monocytes and tumor-associated macrophages (16). However, the present patient refused to undergo liver biopsy after HPD with pembrolizumab and after successful rechallenge with atezolizumab. Thus, important information involving alterations in the expression of interesting genes and individual infiltrating immune cells associated with HPD is absent. Future studies are needed to investigate the potential molecular and cellular mechanisms of HPD and further predictive biomarkers associated with HPD in PD-1/PD-L1 blockade therapy.

### Therapeutic Strategy for HPD

HPD is a rare pattern of tumor progression after ICI therapy, and patients with HPD have poor survival. In a retrospective study involving 406 NSCLC patients treated with PD-1/PD-L1 inhibitors, patients experiencing HPD within the first 6 weeks of treatment with PD-1/PD-L1 inhibitors had a significantly shorter overall survival (OS) than those with only progressive disease (median OS, 3.4 months versus 6.2 months; *P* = 0.003) (25). Patients with HPD usually have worsening symptoms and poor performance status. Patients with HPD according to the TGKR criteria have poorer progression-free survival and OS than those with progressive disease but without HPD (24). Recently, Matos et al. proposed a new method for diagnosing HPD using the RECIST1.1 criteria rather than the TGKR criteria because the definition of HPD according to RECIST1.1 is intuitive and easy to use in daily clinical practice (26).

In general, therapeutic strategies for HPD are extremely limited. After the confirmation of HPD, immunotherapy should be terminated, and patients should switch to other regular or experimental interventions, including radiotherapy, conventional chemotherapy, and antiangiogenic therapy. However, a majority of patients with HPD do not have the opportunity to receive timely subsequent treatment because of rapid clinical deterioration (27). In the KEYNOTE-355 study, progression-free survival was significantly longer among patients with advanced TNBC who received pembrolizumab plus different chemotherapy treatments (paclitaxel protein-bound, or paclitaxel, or gemcitabine plus carboplatin) than among those who received placebo plus chemotherapy (28). However, the present patient developed HPD with pembrolizumab treatment, new chemotherapeutic agents such as sacituzumab govitecan-hziy and eribulin were not approved or available in China. She also had an elevated serum creatinine and progressed on previous treatment with vinorelbine plus carboplatin and nab-paclitaxel plus capecitabine, so different platinum-doublet chemotherapy was not considered prior to an immune checkpoint inhibitor rechallenge. Given that MDM2/MDM4 amplification is a predictive indicator of HPD, MDM2 inhibitors could have potential efficacy in patients with HPD. Fang et al. found that APG-115 plus anti-PD-1 combination therapy leads to enhanced antitumor activity in Trp53wt-, Trp53mut-, and Trp53-deficient syngeneic tumor models. The increases in the number of infiltrated CD8+ T cells and proinflammatory M1 macrophage polarization are linked to the antitumor efficacy of this combination (29). In the present case, rechallenge with atezolizumab was initiated and resulted in a significant objective partial response. This treatment decision was based on the results of the IMpassion130 trial, in which atezolizumab plus nab-paclitaxel prolonged progression-free survival among patients with metastatic TNBC (12), and atezolizumab was available in China at that time. The immunomodulatory effects of chemotherapy, including microtubule-targeting agents, anthracyclines, and antimetabolites, have been identified to expand or activate effector cell populations, inhibit suppressor cell populations, and promote interferon-γ secretion and adaptive PD-L1 expression upregulation (30). Single-cell RNA sequencing showed that there was a large population of PD-1-positive T cells, the number of which significantly decreased after nab-paclitaxel plus pembrolizumab treatment, as well as tissue-resident memory T cells in the responding patient receiving nab-paclitaxel and immunotherapy (31). More recently, a metastatic TNBC patient challenged with pembrolizumab maintained a partial response for over 9 months after failure of initial treatment with atezolizumab plus nab-paclitaxel (32). Rechallenge with another ICI after failure of previous ICI treatment has been reported in other human cancers, such as NSCLC (33–35) and colon cancer (36). In a case series report involving NSCLC, 88.2% of patients received anti‐PD‐1 inhibitor treatment prior to anti‐PD‐L1 inhibitor treatment, and 29.4% received treatment after anti‐PD‐L1 inhibitor treatment; among them, four patients received a third ICI treatment (33). Rechallenge with atezolizumab resulted in an objective partial response in a squamous NSCLC patient who previously experienced disease progression with nivolumab (34). Although the rationale behind this treatment decision was not fully supported by a prospective study, real-world treatment with another ICI monotherapy or in combination with other agent rechallenge could be applied under circumstances without more treatment regimens for advanced cancer patients.

At present, there are no direct comparisons between PD-1 and PD-L1 inhibitors in terms of clinical efficacy. The different targets and immune interactions of PD-1 and PD-L1 inhibitors could affect their antitumor potential in some individuals. One possibility is that T cell influx during pembrolizumab treatment and subsequent *de novo* priming by reactivated dendritic cells (DCs) by atezolizumab contribute to successful rechallenge with ICIs. Although patients treated with anti-PD-L1 antibodies receive limited benefits from retreatment with anti-PD-1 inhibitors (33), expression of PD-L1 seems to predict the efficacy of subsequent ICI switching (35). In fact, PD-L1 expression on tumor cells or on tumor-infiltrating immune cells can independently attenuate anticancer immunity, and PD-L1 on tumor cells can act as a target of PD-L1 inhibitors (37). Durable clinical responses to atezolizumab have been found in advanced NSCLC patients with tumors expressing high levels of PD-L1 on either tumor cells alone or infiltrating immune cells alone (37, 38). Interestingly, CD3+ cells were visible in the pretreatment tumor in this patient, but PD-L1 was not expressed on tumor cells. Moreover, although the PD-1/PD-L1 axis and pathway are typically linked to the activation of T cells, DCs are a crucial target of PD-L1-blocking antibodies. PD-L1 inhibitors relieve B7.1 sequestration in cis through PD-L1 expressed in DCs, which leads to an B7.1/CD28 interaction to increase the priming of T cells (39). It is hypothesized that the introduction of an anti-PD-L1 antibody to a new regimen may reactivate the immune response by targeting DCs.

High TMB is associated with longer survival in metastatic TNBC with ICI therapy (40). Mutations of specific driver genes, including PI3KCA, PI3KCB, DVL3, WWTR1, and ERBB2, exhibit a strong association with immune cell infiltration, suggesting that the pathways that they participate in could be involved in regulating immune cell infiltration (41). Although this patient had low TMB and PD-L1 expression on tumor-infiltrating immune cells was observed, and she harbored a PI3KCA mutation, which could have contributed to the excellent antitumor efficacy of atezolizumab after failure of pembrolizumab. Our previous case report showed that rechallenge with different PD-1/PD-L1 inhibitors was associated with an increased risk of developing high-grade or steroid-resistant pneumonitis, indicating that rechallenge with another PD-1/PD-L1 inhibitor could simultaneously reactivate the immune response in the tumor microenvironment (42). To our knowledge, this is the first report of response to a PD-L1 inhibitor following clinically defined HPD induced by a PD-1 inhibitor in metastatic breast cancer patients. Although there is a lack of conclusive data from prospective trials supporting the efficacy of different ICI retreatment, this approach might be a reasonable option in individual cancer patients who are responsive or not to prior immune checkpoint blockade. Future studies are warranted to determine which patients are most likely to benefit from this strategy of sequential immune checkpoint blockade and to investigate the underlying cellular and molecular mechanisms of successful rechallenge with different ICIs by analyzing the dynamics of the tumor microenvironment, gene expression, and genetic alterations in tumors.

## Data Availability Statement

The original contributions presented in the study are included in the article/supplementary material. Further inquiries can be directed to the corresponding author.

## Ethics Statement

The studies involving human participants were reviewed and approved by The ethics committee, First Affiliated Hospital of Shandong First Medical University. The patients/participants provided their written informed consent to participate in this study. Written informed consent was obtained from the individual(s) for the publication of any potentially identifiable images or data included in this article.

## Author Contributions

DF and YG were involved in the identification and selection of patient cases and drafted the manuscript. ML and BY were involved in the drafting and editing of the manuscript. SH, WZ, JL and YL reviewed and edited the manuscript. JW was involved in the identification, selection, and management of patient cases and reviewed and edited the manuscript. All authors contributed to the article and approved the submitted version.

## Funding

This study was supported by the National Natural Science Foundation of China (Grant No. 81572875), CSCO-MSD Cancer Research Foundation (Grant No. Y-MSD2020-0350), CSCO-PILOT Cancer Research Foundation (Grant No. Y-2019AZMS-0440), Wu Jieping Medical Foundation for Clinical Scientific Research (Grant No. 320.6750.2020-12-16), and the Natural Science Foundation of Shandong Province, China (Grant No. ZR2015PH053).

## Conflict of Interest

The authors declare that the research was conducted in the absence of any commercial or financial relationships that could be construed as a potential conflict of interest.

## Abbreviations

CT, computerized tomography; CTLA-4, cytotoxic T-lymphocyte-associated protein 4; DCs, dendritic cells; EGFR, endothelial growth factor receptor; ER, estrogen receptor; HPD, hyperprogressive disease; ICI, immune checkpoint inhibitor; IHC, immunohistochemistry; irAEs, immune-related adverse events; IVIG, intravenous immunoglobulin; NSCLC, non-small cell lung cancer; OS, overall survival; PD-1, programmed cell death protein-1; PD-L1, programmed death ligand-1; PET/CT, positron emission tomography/computed tomography; PR, progesterone receptor; RECIST, response evaluation criteria in solid tumors; TNBC, triple-negative breast cancer; TGR, tumor growth rate; TGKR, tumor growth kinetics ratio.
